# Diagnostic performance of magnetic resonance imaging in the assessment of periosteal reactions in bone sarcomas using conventional radiography as the reference

**DOI:** 10.1590/0100-3984.2015.0166

**Published:** 2017

**Authors:** José Luiz de Sá Neto, Marcelo Novelino Simão, Michel Daoud Crema, Edgard Eduard Engel, Marcello Henrique Nogueira-Barbosa

**Affiliations:** 1 MSc, Attending Physician in the Department of Radiology and Diagnostic Imaging at the Celso Pierro Maternity Hospital of the Pontifícia Universidade Católica de Campinas (PUC-Campinas), Campinas, SP, Brazil.; 2 PhD, Radiologist at the Hospital das Clínicas da Faculdade de Medicina de Ribeirão Preto da Universidade de São Paulo (HCFMRP-USP), Ribeirão Preto, SP, Brazil.; 3 MD, Radiologist, Musculoskeletal Radiology Service, Hôpital Saint-Antoine, Université Paris IV, Paris, France.; 4 PhD, Professor in the Department of Biomechanics, Medicine, and Rehabilitation of the Locomotor System of the Faculdade de Medicina de Ribeirão Preto da Universidade de São Paulo (FMRP-USP), Ribeirão Preto, SP, Brazil.; 5 Tenured Associate Professor of Radiology and Diagnostic Imaging in the Radiology Division of the Department of Clinical Medicine at the Faculdade de Medicina de Ribeirão Preto da Universidade de São Paulo (FMRP-USP), Ribeirão Preto, SP, Brazil.

**Keywords:** Periosteum, Osteosarcoma, Sarcoma, Ewing, Magnetic resonance imaging, Radiography, Reproducibility of results

## Abstract

**Objective:**

To evaluate the performance of magnetic resonance imaging (MRI) in detecting periosteal reactions and to compare MRI and conventional radiography (CR) in terms of the classification of periosteal reactions.

**Materials and Methods:**

Retrospective study of 42 consecutive patients (mean age, 22 years; 20 men) with a confirmed diagnosis of osteosarcoma or Ewing's sarcoma, MRI and CR images having been acquired pretreatment. Three blinded radiologists detected periosteal reactions and evaluated each periosteal reaction subtype in CR and MRI images: Codman's triangle; laminated; and spiculated. The CR was used as a benchmark to calculate the diagnostic performance. We used the kappa coefficient to assess interobserver reproducibility. A two-tailed Fisher's exact test was used in order to assess contingency between CR and MRI classifications.

**Results:**

In the detection of periosteal reactions, MRI showed high specificity, a high negative predictive value, and low-to-moderate sensitivity. For CR and for MRI, the interobserver agreement for periosteal reaction was almost perfect, whereas, for the classification of different subtypes of periosteal reaction, it was higher for the Codman's triangle subtype and lower for the spiculated subtype. There was no significant difference between MRI and CR in terms of the classifications (*p* < 0.05).

**Conclusion:**

We found no difference between MRI and CR in terms of their ability to classify periosteal reactions. MRI showed high specificity and almost perfect interobserver agreement for the detection of periosteal reactions. The interobserver agreement was variable for the different subtypes of periosteal reaction.

## Abstract (pt)

**Objetivo:**

Avaliar o desempenho da ressonância magnética (RM) na detecção de reação periosteal e comparar a classificação de presença ou ausência de reações periosteais entre a RM e a radiografia convencional (RC).

**Materiais e Métodos:**

Estudo retrospectivo incluindo 42 pacientes consecutivos (idade média, 22 anos; 20 homens) com diagnóstico confirmado de osteossarcoma ou sarcoma de Ewing, tendo exames de RM e RC adquiridos pré-tratamento. Três radiologistas avaliaram às cegas a presença ou ausência de reação periosteal e de cada subtipo de reação periosteal nas imagens de RC e RM: triângulo de Codman, multilamelada e espiculada. A RC foi usada como padrão de referência para cálculo do desempenho diagnóstico. Foi utilizado o coeficiente kappa para reprodutibilidade interobservador. Adicionalmente, foi realizado teste exato de Fisher bicaudal para avaliar se houve diferença significativa entre as leituras da RC e RM.

**Resultados:**

A RM mostrou alta especificidade, alto valor preditivo negativo e baixa sensibilidade na detecção de reação periosteal. A concordância interobservador para a reação periosteal foi quase perfeita para a RC e RM. A concordância interobservador para a classificação dos diferentes subtipos de reação periosteal foi maior para o subtipo triângulo de Codman e menor para o subtipo espiculada. Não houve diferença na detecção por RM e RC (*p* < 0,05).

**Conclusão:**

Não houve diferença significativa entre as classificações da presença ou ausência de reações periosteais entre os métodos RC e RM. A RM apresentou alta especificidade e concordância interobservador quase perfeita para a detecção de reação periosteal. A concordância interobservador para os diferentes subtipos de reação periosteal foi variável.

## INTRODUCTION

Conventional radiology (CR) is the foundation of the initial approach to diseases of the bones and joints, allowing analysis of the biological behavior of focal bone lesions. The identification and characterization of periosteal reactions are part of the evaluation of that behavior and of the degree of aggressiveness of such lesions. It is common to divide periosteal reactions into classical subtypes, and the identification of each of those subtypes can suggest the diagnosis of a disease or specific type of tumor^(1,2)^. In general, biological processes that evolve rapidly or show intense activity result in aggressive forms of periosteal reactions, whereas those resulting from indolent growth processes are nonaggressive^(1-6)^.

Some subtypes of periosteal reaction, such as the solid subtype, are strongly suggestive of nonaggressive, slow-growing lesions, whereas the laminated ("onion skin") subtype suggests processes of intermediate aggressiveness^(7)^. Periosteal reactions that are interrupted, spiculated, or complex suggest aggressive or rapidly growing bone lesions, which have a worse prognosis^(7)^. In practice, however, imaging studies of benign and malignant lesions can reveal overlapping subtypes of periosteal reaction, and the classification of periosteal reactions alone is not sufficient to define the nature or aggressiveness of the bone lesion^(3)^.

Although magnetic resonance imaging (MRI) is considered the best technique for the local staging of musculoskeletal neoplasms^(5-12)^, it is relatively little used in the primary diagnosis of bone tumors and its capacity to evaluate periosteal reactions might be underestimated. The evaluation of periosteal reactions through MRI has rarely been addressed in the medical literature. In two relatively recent review articles on periosteal reactions, both dedicated to the training of radiology residents, the characteristics of periosteal reactions on MRI scans were not analyzed in depth^(1,2)^. Nevertheless, MRI can revealed the periosteal reactions, which appear as lines of low signal intensity in all pulse sequences^(3,8)^.

There have been only a few studies comparing CR and MRI in terms of their performance in the evaluation of periosteal reactions, and those studies were based on the evaluation of MRI scans with low spatial resolution^(13,14)^. In addition, to our knowledge, there have been no studies dedicated specifically to evaluating the reproducibility of imaging methods in the identification and classification of periosteal reactions into subtypes. Given the current importance of MRI in the study of musculoskeletal tumors, there appears to be a gap in the international literature.

The primary objective of this study was to evaluate the diagnostic performance of MRI in the detection of periosteal reactions, using RC as a reference. As a secondary objective, our study aimed to assess the interobserver agreement for MRI and CR in the detection of periosteal reactions.

## MATERIALS AND METHODS

The local human research ethics committee approved the study (Protocol no. 1269/2009). By reviewing the databases containing the radiological and histopathological reports, we identified the patients who had been diagnosed with osteosarcoma or Ewing's sarcoma at our institution. The following inclusion criteria were applied:

1. primary malignant bone neoplasm arising in a long bone;
2. histopathological confirmation;
3. imaging data-from MRI (1.5 T) and CR-acquired prior to treatment and still available;
4. a maximum interval of one week between the CR and MRI studies.

From 2000 to 2014, 42 cases met the inclusion criteria: 8 cases of Ewing's sarcoma; and 34 cases of osteosarcoma. The mean age of the patients was 22 years. Of the 42 patients evaluated, 20 were male and 22 were female.

**Figure 1:**
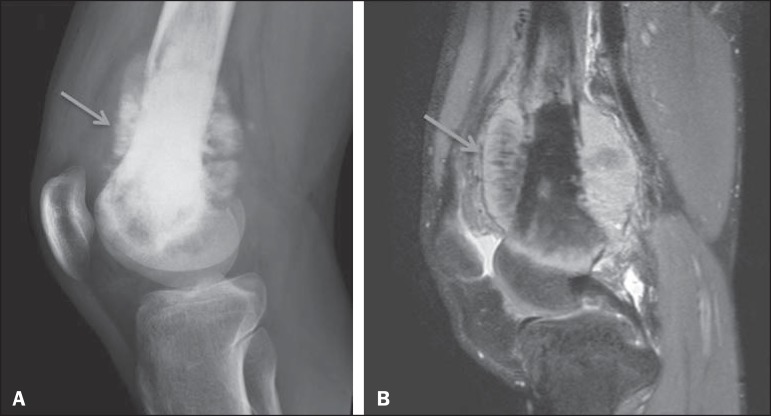
Osteosarcoma of the distal femur presenting a spiculated periosteal reaction, well demonstrated by CR (arrow in **A**) and MRI (arrow in **B**).

**Figure 2:**
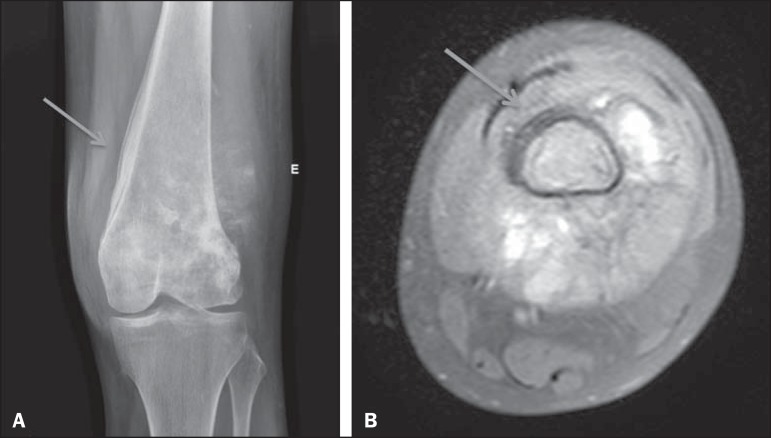
Osteosarcoma of the femur with extension to the soft tissues. Laminated periosteal reaction identified on CR (arrow in **A**). Although the laminated reaction was not identified by two of the three observers, it is possible to observe it retrospectively in the MRI study (arrow in **B**).

**Figure 3:**
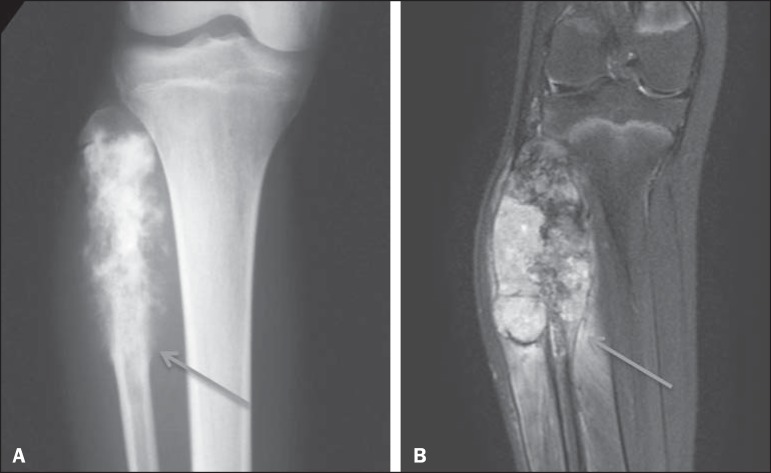
Codman's triangle, identified by the observers only on the MRI scan (arrow in **B**). Retrospective evaluation showing an outline of an interrupted periosteal reaction also on CR (arrow in **A**).

Three musculoskeletal radiologists, all of whom were blinded to the diagnoses, working independently, retrospectively identified periosteal reactions. The radiologists also classified each case as meeting the criteria for one of the three major subtypes of aggressive periosteal reactions seen on CR and MRI: spiculated (Figure 1); laminated ("onion skin") (Figure 2); and Codman's triangle (Figure 3). The same radiologist read the MRI and CR images twice, with an interval of at least three months between the two readings. In the classification of the aggressive periosteal reactions by subtype, it was accepted that the same case could involve more than one of the subtypes evaluated. Using the readings of the three radiologists, we evaluated interobserver agreement with the kappa statistic (κ), for the identification of periosteal reactions in general and for that of each specific subtype. The interpretation of the κ values obtained was based on the following pattern: κ < 0, no agreement; 0 ≤ κ ≤ 0.20, no to slight agreement; 0.21 ≤ κ ≤ 0.40, fair agreement; 0.41 ≤ κ ≤ 0.60, moderate agreement; 0.61 ≤ κ ≤ 0.80, substantial agreement; and 0.81 ≤ κ ≤ 1, almost perfect agreement^(15)^.

Consensus readings were used in order to quantify the diagnostic performance of MRI in the detection of periosteal reactions and of each subtype of aggressive periosteal reaction. The consensual classifications of the CR and MRI readings were obtained from the initial classifications provided by the three radiologists. When there was agreement between the three readings, the consensual classification corresponded to the classification of the three radiologists. The periosteal reactions and specific periosteal reaction subtypes were classified in a dichotomous manner (as present or absent). Therefore, when there was any disagreement, the final (consensus) classification was that of the two radiologists who agreed (i.e., the majority opinion, two against one, prevailed). The consensus classification of CR images was obtained in a manner analogous to that of MRI scans and served as the reference for quantifying the diagnostic performance.

A two-tailed Fisher's exact test was conducted in order to determine whether there was a statistically significant difference between the CR and MRI readings. The level of statistical significance was set at a < 0.05.

## RESULTS

The interobserver agreement for CR and MRI analysis is presented in Tables 1 and 2, respectively. In general, the interobserver agreement for the detection of a periosteal reaction was almost perfect for the CR analysis and substantial to almost perfect for the MRI analysis.

**Table 1:** Interobserver agreement for the detection of periosteal reactions and for the classification of periosteal reactions by specific subtype from conventional radiography images, showing the mean values obtained for the kappa coefficient and 95% confidence intervals.

Interobservador agreement – Conventional radiography						
	Observers 1 and 2		Observers 1 and 3		Observers 2 and 3	
Feature	Kappa	95% CI	Kappa	95% CI	Kappa	95% CI
Periosteal reaction		1	0.88	0.72–1.0	0.88	0.72–1.0
Codman’s triangle subtype	0.51	0.15–0.87	0.88	0.65–1.0	0.42	0.08–0.77
Laminated subtype	0.33	0.0–0.84	0.26	0.0–0.89	0.67	0.25–1.0
Spiculated subtype	0.16	0.0–0.53	0.2	0.0–0.6	0.86	0.61–1.0

95% CI, 95% confidence interval.

**Table 2:** Interobserver agreement for the detection of periosteal reactions and for the classification of periosteal reactions by specific subtype from magnetic resonance imaging scans, showing the mean values obtained for the kappa coefficient and 95% confidence intervals.

Interobservador agreement – Magnetic resonance imaging						
	Observers 1 and 2		Observers 1 and 3		Observers 2 and 3	
Feature	Kappa	95% CI	Kappa	95% CI	Kappa	95% CI
Periosteal reaction	0.88	0.72–1.0	0.76	0.54–0.98	0.64	0.0–1.0
Codman’s triangle subtype	0.54	0.14–0.93	0.76	0.45–1.0	0.54	0.14–0.93
Laminated subtype	0.76	0.54–0.98	0.76	0.54–0.98	0.43	0.0–1.0
Spiculated subtype	0.17	0.0–0.58	0.39	0.0–0.91	0.55	0.16–0.93

95% CI, 95% confidence interval.

Tables 3 and 4 show, respectively, the results and the diagnostic performance of MRI, with the CR as the reference. For the detection of periosteal reactions, MRI showed high specificity and a high negative predictive value, whereas it showed only moderate sensitivity and a moderate positive predictive value. For the identification of the specific subtypes of aggressive periosteal reaction, MRI showed substantial sensitivity and moderate specificity.

**Table 3:** Results for magnetic resonance imaging in the detection of periosteal reactions in general and in the classification of periosteal reactions by subtype, with conventional radiography as the reference.

Feature	TP	FP	FN	TN
Periosteal reaction	36	1	3	2
Codman’s triangle subtype	5	4	3	30
Laminated subtype	4	2	8	28
Spiculated subtype	5	6	4	27

TP, true-positive; FP, false-positive; FN, false-negative; TN, true-negative.

**Table 4:** Diagnostic performance of magnetic resonance imaging in the detection of periosteal reactions in general and in the classification of aggressive periosteal reactions by subtype, using that of conventionar radiography as the reference.

Feature	Positive predictive value	Negative predictive value	Sensitivity (95% CI)	Specificity (95% CI)
Periosteal reaction	0.66	0.92	0.4 (0.05–0.85)	0.97 (0.85–0.99)
Codman’s triangle subtype	0.91	0.55	0.88 (0.72–0.96)	0.62 (0.25–0.91)
Laminated subtype	0.93	0.33	0.78 (0.6–0.9)	0.66 (0.22–0.95)
Spiculated subtype	0.81	0.55	0.87 (0.7–0.96)	0.45 (0.16–0.76)

95% CI, 95% confidence interval.

The Fisher's exact test showed no statistical differences between CR and MRI for the identification of periosteal reactions in general (*p* = 0.033); Codman's triangle type periosteal reactions (*p* = 0.006); laminate periosteal reactions (*p* = 0.046); and spiculated periosteal reactions (*p* = 0.038).

## DISCUSSION

The evaluation of the musculoskeletal system by imaging methods has been the motivation for a series of recent studies in the radiology literature of Brazil^(16-23)^.

The clinical approach to the analysis of bone lesions includes the identification of periosteal reactions and their classification into subtypes. In general, the identification of the periosteal reaction subtype can help the radiologist characterize bone lesions as more aggressive or more indolent. Osteosarcoma and Ewing's sarcoma are two of the most common primary malignant bone tumors, occurring predominantly in young patients, and can be associated with various subtypes of aggressive periosteal reaction^(24-27)^.

There have been pictorial essays of a didactic nature that have illustrated subtypes of periosteal reactions and how they can be identified through MRI^(4,28)^. However, those studies did not make statistical comparisons between CR and MRI or conduct objective evaluations of the diagnostic performance of MRI in the detection of periosteal reactions.

We identified two English-language articles comparing CR and MRI in terms of their roles in the evaluation of periosteal reactions in bone tumors^(13,14)^. One of those studies was published in 1987 and involved MRI sequences acquired in a 0.15 T scanner; the results were therefore based on the evaluation of images of a quality inferior to that of those currently available^(13)^. The authors (radiologists) qualitatively assessed the detection of periosteal reactions by both imaging techniques, applying a score of 1 to 5, in which a higher score indicated an image that was more appropriate for the evaluation of the periosteal reactions^(13)^. They concluded that MRI scans were inferior to the CR images in relation to the evaluation of periosteal reactions^(13)^. Because the MRI scans were acquired at low spatial resolution, it might not be appropriate to extrapolate these results to the high-resolution MRI scans currently available. The other study also compared CR and MRI in terms of their performance in the evaluation of periosteal reactions in osteosarcomas^(14)^. The authors of that study evaluated 54 patients with a histologically confirmed diagnosis of osteosarcoma and classified the periosteal reactions as absent, laminated, spiculated, mixed (laminated and spiculated), or Codman's triangle. The MRI scans included in their study were also acquired at a low (0.5 T) magnetic field. The evaluations were carried out in consensus, and the interobserver agreement was therefore not evaluated. Another limitation of the second study was the way in which the CR images and MRI scans were compared. The only parameter used was the k coefficient of agreement between the CR and MRI readings, which seems inappropriate for the comparison of the performance of two diagnostic imaging techniques. The authors did not calculate the sensitivity and specificity for the detection of periosteal reactions and their subtypes^(14)^.

Our results show that the diagnostic performance of MRI in the detection of periosteal reactions in bone sarcomas was satisfactory and comparable to that of CR. In comparison with CR, MRI showed higher specificity and a higher negative predictive value in the detection of periosteal reactions. However, the sensitivity of MRI for the detection of periosteal reaction could be considered relatively low.

To our knowledge, the present study was the first to be dedicated to the analysis of interobserver agreement for the MRI and CR detection of periosteal reactions in bone tumors. We also believe that this was the first study of interob server agreement for the MRI and CR classification of aggressive periosteal reactions by subtype. Our results indicate that the two diagnostic imaging methods showed near-perfect interobserver agreement for the detection of periosteal reactions and substantial interobserver agreement for the identification of Codman's triangle. For the identification of the spiculated and laminated subtypes of periosteal reaction, in general, CR showed lower interobserver agreement than did MRI, especially for the laminated subtype.

Overall, we found no statistically significant difference between CR and MR for the detection of periosteal reactions (*p* < 0.05). We also found that the two imaging methods did not differ significantly in terms of their utility in the classification of aggressive periosteal reactions by subtype (*p* < 0.05).

The present study has some limitations that should be noted. First, it was a retrospective study. In addition, the MRI acquisition protocols were not standardized, varying during the study period. Another limitation is our use of CR as the reference to assess the diagnostic performance of MRI, because it is possible that CR is not the ideal reference, given that periosteal reactions initially have non-mineralized components that are not detectable by CR. An experimental study of osteomyelitis, using histology as the reference, showed that MRI was more sensitive than were CR and computed tomography in the detection of periosteal reactions^(29)^. In the present study, it would not have been possible to use histopathology as a reference, because, although biopsy samples were evaluated in all cases, no details were available on the presence and formation of periosteal reactions in volumetric form. Nevertheless, periosteal reactions are routinely evaluated by CR in clinical practice, and that is why we chose to use CR as the reference in our study.

## CONCLUSIONS

There was no significant difference between CR and MRI in terms of their utility in detecting periosteal reactions. Our results suggest that there is high interobserver agreement between the two methods for the detection of periosteal reactions. The interobserver agreement between CR and MRI for the classification of aggressive periosteal reactions by subtype was variable, being better for the identification of the Codman's triangle subtype, whereas it was worse for the laminated and spiculated subtypes. For the diagnosis of periosteal reaction, with CR as a reference, MRI showed high specificity and low sensitivity.
